# A mindfulness-based intervention to increase resilience to stress in university students (the Mindful Student Study): a pragmatic randomised controlled trial

**DOI:** 10.1016/S2468-2667(17)30231-1

**Published:** 2017-12-19

**Authors:** Julieta Galante, Géraldine Dufour, Maris Vainre, Adam P Wagner, Jan Stochl, Alice Benton, Neal Lathia, Emma Howarth, Peter B Jones

**Affiliations:** a Department of Psychiatry, University of Cambridge, Cambridge, UK; b University Counselling Service, University of Cambridge, Cambridge, UK; c Educational and Student Policy, Academic Division, University of Cambridge, Cambridge, UK; d National Institute for Health Research Collaboration for Leadership in Applied Health Research and Care East of England, Cambridge, UK; e British Association for Counselling and Psychotherapy: Universities and Colleges Division, Leicestershire, UK; f Norwich Medical School, University of East Anglia, Norwich Research Park, Norwich, UK; g Department of Computer Science, University College London, London, UK

## Abstract

**Background:**

The rising number of young people going to university has led to concerns about an increasing demand for student mental health services. We aimed to assess whether provision of mindfulness courses to university students would improve their resilience to stress.

**Methods:**

We did this pragmatic randomised controlled trial at the University of Cambridge, UK. Students aged 18 years or older with no severe mental illness or crisis (self-assessed) were randomly assigned (1:1), via remote survey software using computer-generated random numbers, to receive either an 8 week mindfulness course adapted for university students (Mindfulness Skills for Students [MSS]) plus mental health support as usual, or mental health support as usual alone. Participants and the study management team were aware of group allocation, but allocation was concealed from the researchers, outcome assessors, and study statistician. The primary outcome was self-reported psychological distress during the examination period, as measured with the Clinical Outcomes in Routine Evaluation Outcome Measure (CORE–OM), with higher scores indicating more distress. The primary analysis was by intention to treat. This trial is registered with the Australia and New Zealand Clinical Trials Registry, number ACTRN12615001160527.

**Findings:**

Between Sept 28, 2015, and Jan 15, 2016, we randomly assigned 616 students to the MSS group (n=309) or the support as usual group (n=307). 453 (74%) participants completed the CORE–OM during the examination period and 182 (59%) MSS participants completed at least half of the course. MSS reduced distress scores during the examination period compared with support as usual, with mean CORE–OM scores of 0·87 (SD 0·50) in 237 MSS participants versus 1·11 (0·57) in 216 support as usual participants (adjusted mean difference −0·14, 95% CI −0·22 to −0·06; p=0·001), showing a moderate effect size (β −0·44, 95% CI −0·60 to −0·29; p<0·0001). 123 (57%) of 214 participants in the support as usual group had distress scores above an accepted clinical threshold compared with 88 (37%) of 235 participants in the MSS group. On average, six students (95% CI four to ten) needed to be offered the MSS course to prevent one from experiencing clinical levels of distress. No participants had adverse reactions related to self-harm, suicidality, or harm to others.

**Interpretation:**

Our findings show that provision of mindfulness training could be an effective component of a wider student mental health strategy. Further comparative effectiveness research with inclusion of controls for non-specific effects is needed to define a range of additional, effective interventions to increase resilience to stress in university students.

**Funding:**

University of Cambridge and National Institute for Health Research Collaboration for Leadership in Applied Health Research and Care East of England.

## Introduction

Supporting young people's health and wellbeing is an investment that results in considerable economic benefit.^1^ Participation in higher education is growing among young people, including students from increasingly diverse backgrounds; more than a third of each generation now attends university in England.^2^ Prevalence of mental illness in first-year undergraduates is lower than in the general population, but becomes higher during the second year.^3^ The number of students accessing university counselling in some services in the UK grew by 50% from 2010 to 2015, surpassing the growth in the number of university entrants in the same period.^4^ Reasons for this increase are unclear, with little consensus about whether students are experiencing more mental disorders, are less resilient than in the past, whether there is less stigma in accessing support, and how all these factors affect academic attainment.^5^, ^6^, ^7^ Nevertheless, the journey through university provides a golden, yet underused, opportunity for prevention of mental illness in young people.^8^

Mindfulness is a means of training the regulation of attention for the purpose of mental health promotion, and has become popular in universities.^9^ Uptake of the approach might partly be explained by the perception of mindfulness training as a skill rather than a mental health intervention.^10^ Evidence has shown the efficacy of mindfulness training in improvement of symptoms of common mental disorders, such as anxiety and depression.^11^ However, little robust evidence exists for the effectiveness in prevention of common mental disorders in university students, and no studies have actively monitored adverse effects. Previous trials focused mostly on health-care students, but most were underpowered and had no prospective protocol or clear primary outcome. Furthermore, there are concerns about multiple testing, researcher allegiance bias with teachers acting as researchers, inadequate analysis and treatment of missing data, and unrealistically short follow-up times.^12^, ^13^, ^14^ One good-quality study^15^ randomly assigned 288 health-care students to receive mindfulness training or be placed on a waiting list and found moderate post-intervention effects on psychological distress and wellbeing. A systematic review and meta-analysis^16^ assessed findings from nine randomised and non-randomised trials and showed that mindfulness reduced anxiety among university students.

**Research in context**

**Evidence before this study**

On March 20, 2017, we searched CENTRAL, CINAHL, Embase, MEDLINE, and PsycINFO, with the terms “mindfulness” and “meditation”, combined with “university”, “college”, “school”, “higher education”, “postgraduate”, “undergraduate”, “student”, or “trainee”, with no date or language restrictions. Our search identified comprehensive systematic reviews and meta-analyses showing evidence for the efficacy of mindfulness meditation programmes in improvement of symptoms of common mental disorders, such as depression or anxiety. Systematic reviews of mindfulness training for university students show preliminary evidence for its effectiveness. However, the evidence is inconsistent and mostly from non-randomised evaluations or evaluations involving only health-care students. Previous randomised controlled trials of mindfulness support for university students have been generally underpowered, enrolled too few students, and had no prospective protocol, no primary outcome, multiple testing problems, researcher allegiance bias, inadequate analysis or treatment of missing data, lack of follow-up, or other methodological and reporting issues. The largest and best quality pre-existing trial randomly assigned 288 health-care students to mindfulness-based stress reduction or a wait-list control group. The findings showed moderate post-intervention effects on psychological distress and wellbeing.

**Added value of this study**

We present a randomised controlled trial of provision of an 8 week Mindfulness Skills for Students (MSS) course in the year leading up to the main annual examination period. Compared with participants assigned to receive mental health support as usual, MSS participants were a third less likely to experience psychological distress at a clinically relevant level during the examination period. Of the 30 students in each MSS course, an average of five students will be prevented from experiencing clinical levels of distress during examinations—evidence of an effective preventive intervention. The absence of a control for non-specific effects precludes us from attributing our findings entirely to the specific components of mindfulness training, but evidence of such effects is available in the published literature. This trial, co-produced with students and university officers, is an example of participatory research informing student welfare policy.

**Implications of all the available evidence**

Evidence indicates that preventive mindfulness courses are acceptable to students and universities, and are feasible and effective components of a wider student mental health strategy. Comparative effectiveness research is needed into preventive mental health interventions for students.

In view of the increasing demands on student mental health services, the popularity of mindfulness, and its promise as a preventive intervention, we did the Mindful Student Study to assess whether provision of mindfulness courses to university students would improve their resilience to stress. Our primary hypothesis was that provision of mindfulness courses would reduce students' psychological distress during the examination period, when stress peaks,^7^ compared with support as usual. A reduction in distress while under a universal stressor (examinations) was deemed an indicator of resilience to stress.

## Methods

### Study design and participants

We did this pragmatic randomised controlled trial at the University of Cambridge, UK. Eligibility criteria were self-assessed by students, and replicated those used routinely by the University of Cambridge Counselling Service. We included current undergraduate or postgraduate students (aged ≥18 years) at the University of Cambridge, and students who believed they could attend at least seven of the eight sessions of the mindfulness course. We excluded students who were currently experiencing severe periods of anxiety or depression; a severe mental illness, such as hypomania or psychotic episodes; recent bereavement or major loss; or any other serious mental or physical health problem that would affect their ability to engage with the course.

**Figure 1:**
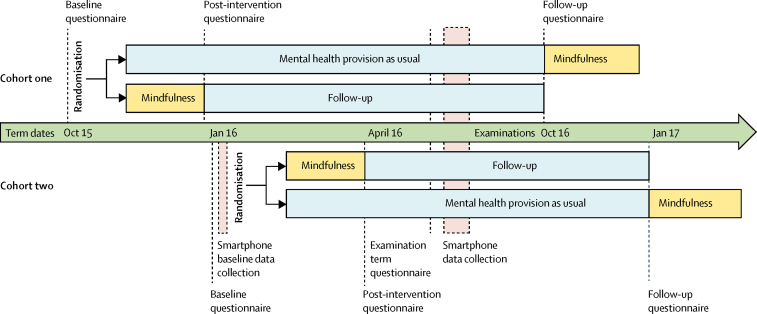
Participant timeline^17^ Term dates bar is not to scale.

Participants were recruited in two waves before randomisation. The first wave promoted the study and enrolled interested students in the term starting in October, 2015 (cohort one). The second wave promoted the study again and enrolled interested students in the term starting in January, 2016 (cohort two; figure 1).

The study protocol^17^ was submitted to the Australian New Zealand Clinical Trials Registry on Aug 31, 2015, before the study began, and accepted on Oct 30, 2015. The Cambridge Psychology Research Ethics Committee approved the trial on Aug 25, 2015 (number PRE.2015.060). We set up an independent data monitoring and ethics committee, and co-produced the trial with students and university officers to increase the validity of the results. All participants provided written informed consent.

### Randomisation and masking

The study management team enrolled participants and sent them personal links to an online baseline questionnaire. After completion of the questionnaire, participants were randomly assigned (1:1), via remote survey software (Qualtrics) using computer-generated random numbers (simple randomisation), to receive either mindfulness training with the Mindfulness Skills for Students (MSS) course plus mental health support as usual, or mental health support as usual alone.

Each participant was informed of their allocation automatically after completion of the baseline questionnaire. Concurrently, members of the study management team were also informed automatically of participants' allocation; thus, the allocation process was concealed from the researchers. Due to the nature of the intervention, participants were aware of group allocation for the duration of the study. Data collection was remote and automatic using the web-based Qualtrics software to ensure masking of outcome assessors. The primary analysis was done by a statistician masked to group assignment.

### Procedures

The MSS intervention consisted of a secular, face-to-face, group-based skills training programme based on the course book “Mindfulness: a practical guide to finding peace in a frantic world”,^18^ and adapted for university students (appendix p 2).^17^ Adaptations were focused on permeating every session with elements of flexibility, self-discovery, self-compassion, and empowerment, aimed at generating a natural transfer of skills developed in meditation to study, decision making, and relationships. The course aimed to optimise wellbeing and resilience for all students, and was not specifically developed for those with distress in a clinical range.

Seven MSS courses ran in parallel during university terms, with up to 30 students in each course, all delivered by an experienced and certified mindfulness teacher. The eight, weekly sessions lasted 75–90 min. Sessions included mindfulness meditation exercises, periods of reflection and inquiry, and interactive exercises. Students were encouraged to also practise at home. The recommended home practise time varied throughout the course, starting at 8 min and increasing to about 15–25 min per day. Home practise included meditations from the course book's audio files and other mindfulness practices, such as a mindful walk, mindful eating, and habit breakers. Before and after each class, students received a generic email from the mindfulness teacher with relevant materials.

Students were required to choose a usual session time and day to attend each week, but when this was not possible, students could attend an alternative session within the same week (so-called session hopping). Students were contacted by email when they missed a session to check whether the absence was related to a negative experience with mindfulness. Students were also given the opportunity to talk with the teacher in confidence outside course times if they had any problems, or needed clarification. This routine practice ran for two terms before and then during the trial.

Support as usual consisted of access to comprehensive centralised support at the University of Cambridge Counselling Service in addition to support available from the university and its colleges, and from health services external to the University, including the National Health Service (NHS). Participants assigned to receive support as usual were guaranteed a space in the mindfulness courses in the following year, and were requested to inform the team if they decided to learn mindfulness elsewhere during the follow-up period.

MSS courses were free to students. £11 was available to each participant as a token of appreciation for questionnaire completion.^17^

### Outcomes

The primary outcome was psychological distress, measured with the Clinical Outcomes in Routine Evaluation Outcome Measure (CORE–OM), during the main annual examination period, as defined by the Student Registry.^17^ This period spans May 16, 2016, to June 10, 2016—the most stressful weeks for most students. Participants in cohort one started the course in October, 2015, and those in cohort two in January, 2016. CORE–OM is a 34 item scale that has been widely used in UK university students.^19^ Higher scores indicate more distress. The total mean score (range 0–4) is obtained by dividing the total score by the number of completed items (as long as no more than three items have been missed).^20^

Prespecified secondary outcomes were CORE–OM score immediately after the MSS courses (post intervention); scores on the 14 item Warwick–Edinburgh Mental Wellbeing Scale post-intervention and during the examination period^21^ (total score is calculated by adding the response values of all items [range 14–70, higher scores indicate greater wellbeing]); examination results graded according to the British undergraduate degree classification system (examination ranking was unavailable); numbers of requests for special examination arrangements for any cause, and due to mental health problems; inability to sit examinations; questions assessing the perceived effect of problems on academic performance (“To what extent are you considering leaving your course?”, “To what extent do you have problems affecting your study?”, and “To what extent do you have problems affecting your overall experience at University/College?”); daily questions assessing problem-focused and emotion-focused coping with academic workload for 2 weeks during the examination period (appendix p 5);^17^, ^22^ physical activity patterns (diurnal and sleep; smartphone accelerometer data were automatically sampled every 15 min for 2 weeks during the examination period [appendix p 6]); and a measure of altruism, based on offering high-street shopping vouchers to participants upon questionnaire completion (equivalent to £3 for post-intervention and £5 for examination period questionnaires), with a choice to donate them to a named charity. Prespecified secondary outcomes at 1 year follow-up are yet to be analysed and will be reported elsewhere. We additionally collected information about process measures (eg, MSS attendance).^17^

During the study, we actively and systematically monitored for adverse events,^23^ using three different routes for identification, as detailed in the study protocol.^17^ Emergence of symptoms was recorded on a form sent to the independent data monitoring and ethics committee, who determined whether they could be related to the intervention. Participants with adverse events were offered support.^17^

### Statistical analysis

Data collection began 2 weeks before the start of the examination period—about 6 months after randomisation for participants in cohort one, and 3 months after randomisation for those in cohort two (figure 1). To detect a change in CORE–OM score of SD 0·3 at a p value of less than 0·05 with 90% power, 550 students were estimated to be needed, allowing for 20% loss to follow-up.^20^ Any clustering effect within each course delivery was expected to be negligible: although this is a group intervention, the work is highly personal, all the courses were taught by the same teacher, each mindfulness course included students from different colleges and academic courses, and the session-hopping option introduced some variability. However, we did intraclass correlation analyses of outcome and attendance data to measure the extent of clustering.

The primary analysis was by intention to treat. Regression modelling of imputed data included baseline scores, sex, age, and timing of receipt of intervention relative to exams as covariates.^24^ Multiple imputation addressed missing data (appendix pp 3, 4). We did post-hoc two-sample *t* tests, and prespecified per-protocol (minimum dose assumed to be 50% attendance of sessions) and subgroup analyses (interaction tests) of the primary outcome. A post-hoc exploratory subgroup analysis was added to test the influence of being in cohort one versus cohort two. Normative UK data were compared with baseline values (prespecified) and primary outcome data (post hoc).^20^ We considered standardised effect sizes as small (Cohen's *d* 0·2), moderate (0·5), or large (0·8).^25^

Regression modelling of the imputed datasets was used for the secondary outcomes of post-intervention CORE–OM and Warwick–Edinburgh Mental Wellbeing Scale scores; no data were imputed for the other, more exploratory, outcomes. A total daily coping score was plotted by intervention group, together with locally weighted scatterplot smoothing with 95% CIs (appendix p 5). Physical activity scores, derived from accelerometer data (appendix p 6), were calculated from the magnitude of the acceleration vector.^26^ Aggregated physical activity scores were categorised according to time of day to assess diurnal and nocturnal movement, and plotted with locally weighted scatterplot smoothing and confidence intervals. Odds ratios were calculated for binary outcomes and χ^2^ and Fisher's exact tests for ordinal outcomes (including examination grades because no student had more than one). We used logistic regression to assess baseline predictors of whether the primary outcome was completed. Analyses were done at a two-sided α level of 0·05, using R (version 3.3.2).

This trial is registered with the Australian and New Zealand Clinical Trials Registry, number ACTRN12615001160527.

### Role of the funding source

The funders of the study had no role in study design, data collection, data analysis, data interpretation, or writing of the report. The corresponding author had full access to all of the data in the study and had final responsibility for the decision to submit for publication.

## Results

**Figure 2:**
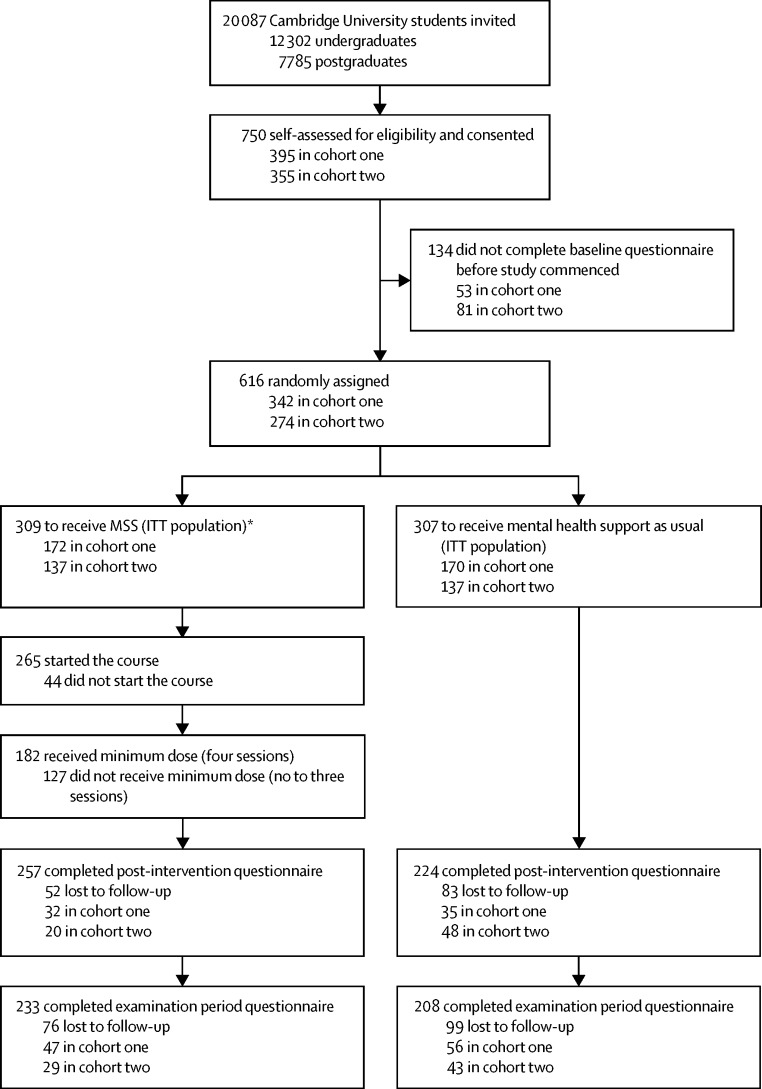
Trial profile MSS=Mindfulness Skills for Students. ITT=intention-to-treat. *Intervention providers comprised one mindfulness teacher, one administrative team, and one centre in which the intervention was done.

Between Sept 28, 2015, and Jan 1, 2015, we randomly assigned 616 students to the MSS group (n=309) or the support as usual group (n=307; figure 2). Five (2%) people, all in the support as usual group, withdrew from the study; three of four in their final year said this was because they could not undertake the MSS course the following year. 182 (59%) participants attended four or more MSS sessions (figure 2). 39 (13%) participants provided reasons for abandoning their mindfulness course; schedule conflicts (n=16) and being too busy (n=12) were the most frequent reasons. 15 (5%) participants cancelled without attending any sessions (appendix p 6). 26 (8%) participants in the support as usual group practised more than 10 h of any type of meditation or did an 8 week mindfulness course after randomisation; data for meditation practice were unavailable for 114 (37%) participants (no response).

Baseline characteristics were similar between groups (table 1). Higher wellbeing at baseline and final-year status reduced the likelihood of completing the primary outcome measure (appendix p 11). The intraclass correlation coefficient for the main outcome was 0, and for the other outcomes ranged from 0 to 0·016; therefore, outcomes are reported without correction for clustering effect.^27^

**Table 1:** Baseline characteristics

		**MSS group (n=309)**	**Support as usual group (n=307)**
Sex			
	Female	187 (61%)	201 (65%)
	Male	122 (39%)	106 (35%)
Age (years)			
	17–21	139 (45%)	140 (46%)
	22–30	144 (47%)	142 (46%)
	≥31	23 (8%)	24 (8%)
UK or European Union nationality*		239 (78%)	231 (76%)
Ethnic origin			
	Asian	51 (17%)	67 (23%)
	Black	4 (1%)	2 (1%)
	Mixed	17 (6%)	23 (8%)
	Other	9 (3%)	7 (2%)
	White	214 (73%)	194 (66%)
Disability		35 (11%)	39 (13%)
Degree level			
	Undergraduate	164 (53%)	163 (53%)
	Taught masters	28 (9%)	27 (9%)
	Research masters	35 (11%)	39 (13%)
	PhD	82 (27%)	78 (25%)
Year			
	1	150 (49%)	147 (48%)
	2	64 (21%)	73 (24%)
	3	69 (22%)	54 (18%)
	4	18 (6%)	28 (9%)
	5	7 (2%)	4 (1%)
	6	0	1 (<1%)
Final year		121 (39%)	122 (40%)
Department			
	Arts and humanities	58 (19%)	62 (20%)
	Biological sciences	65 (21%)	59 (19%)
	Clinical medicine	14 (5%)	16 (5%)
	Humanities and social sciences	104 (34%)	94 (31%)
	Physical sciences	32 (10%)	30 (10%)
	Technology	36 (12%)	46 (15%)
Previous meditation†		27 (10%)	36 (13%)

Data are n (%). MSS=Mindfulness Skills for Students.

* Inferred from whether participants paid their study fees according to UK or European Union rates, or overseas rates.

† Spent in total more than 10 h meditating in the past, or completed an 8 week mindfulness course.

453 (74%) participants completed the CORE–OM during the examination period (table 2). MSS reduced distress during the examination period compared with support as usual: participants' distress scores were on average 0·25 CORE–OM total mean score points lower in the MSS group than in the support as usual group after adjustment for our a-priori set of covariates (table 2, table 3, appendix p 14). In standardised terms, this difference is an average of 0·44 SDs (95% CI 0·60–0·29; p<0·0001)—a moderate effect size (appendix p 14).^25^ Sensitivity and per-protocol analyses gave similar results (p<0·0001 in all cases; appendix p 8).

**Table 2:** Baseline, post-intervention and exam period distress (CORE-OM) and wellbeing (WEMWBS) scores

	**Baseline**			**Post-intervention**			**Examination period**		
	n	Mean (SD)	Median (min–max)	n	Mean (SD)	Median (min–max)	n	Mean (SD)	Median (min–max)
**CORE–OM**									
Support as usual	305	0·97 (0·51)	0·91 (0·00–2·79)	227	1·04 (0·54)	0·94 (0·00–2·82)	216	1·11 (0·57)	1·06 (0·09–3·15)
MSS	309	1·01 (0·54)	0·91 (0·03–2·97)	255	0·88 (0·53)	0·76 (0·06–2·71)	237	0·87 (0·50)	0·79 (0·03–3·06)
Total	614*	0·99 (0·53)	0·91 (0·00–2·97)	482	0·96 (0·54)	0·82 (0·00–2·82)	453	0·98 (0·55)	0·94 (0·03–3·15)
**WEMWBS**									
Support as usual	307	48·61 (8·50)	48·00 (26·00–70·00)	221	46·87 (9·01)	47·00 (28·00–70·00)	214	46·36 (9·05)	46·50 (20·00–70·00)
MSS	307	48·01 (8·58)	48·00 (17·00–70·00)	254	49·61 (8·88)	50·50 (20·00–70·00)	235	48·90 (9·04)	49·00 (14·00–70·00)
Total	614*	48·31 (8·54)	48·00 (17·00–70·00)	475	48·34 (9·03)	49·00 (20·00–70·00)	449	47·69 (9·13)	48·00 (14·00–70·00)

CORE–OM=Clinical Outcomes in Routine Evaluation Outcome Measure. MSS=Mindfulness Skills for Students. WEMWBS=Warwick-Edinburgh Mental Wellbeing Scale.

* For each questionnaire, two students replied “prefer not to answer” to some items, failing to provide enough answers to calculate a baseline score.

**Table 3:** Outcomes comparing MSS (reference) with support as usual

		**Timepoint**	**Result**
CORE–OM (n=482)		Post-intervention	Favours MSS, adjusted mean difference −0·14, 95% CI −0·22 to −0·06; p=0·001
CORE–OM (n=453)*		Examination period	Favours MSS, adjusted mean difference −0·25, 95%CI −0·34 to −0·16; p<0·0001 (appendix p 14)
WEMWBS (n=475)		Post-intervention	Favours MSS, adjusted mean difference 2·75, 95% CI 1·42 to 4·09; p=0·0001
WEMWBS (n=449)		Examination period	Favours MSS, adjusted mean difference 2·61, 95% CI 1·12 to 4·10; p=0·001
Exam grades (n=292)		Examination period	Non-linear relationship; Fisher's exact p=0·04 (appendix p 15)
Requests for special examination arrangements (n=415)			
	For any issue	Examination period	OR 0·70, 95% CI 0·37 to 1·30; p=0·24 (appendix p 16)
	For mental health issues	Examination period	OR 0·72, 95% CI 0·10 to 4·28; p=0·72 (appendix p 16)
Intermissions of study (n=616)		Examination period	Inability OR 1·67, 95% CI 0·32 to 10·82; p=0·72 (appendix p 17)
Considering leaving course (n=447)		Examination period	χ^2^ 3·65, four degrees of freedom; p=0·46 (appendix p 18)
Problems affecting study (n=449)		Examination period	MSS participants had fewer problems, χ^2^ 10·26, four degrees of freedom; p=0·04 (appendix p 18)
Problems affecting experience (n=448)		Examination period	MSS participants had fewer problems, χ^2^ 11·28, four degrees of freedom; p=0·02 (appendix p 18)
Day-to-day coping (n=191)		Examination period	No differences between groups (appendix p 32)
Physical activity (n=31)		Examination period	MSS participants had less afternoon activity (appendix p 31)
Altruism (n=479)		Post-intervention	Donation OR 1·95, 95% CI 1·34 to 2·86; p=0·0003 (appendix p 19)
Altruism (n=450)		Examination period	Donation OR 1·80, 95% CI 1·22 to 2·66; p=0·003 (appendix p 19)

MSS=Mindfulness Skills for Students. CORE–OM=Clinical Outcomes in Routine Evaluation Outcome Measure. WEMWBS=Warwick–Edinburgh Mental Wellbeing Scale. OR=odds ratio.

* Primary outcome measure.

To explore the practical effect of this primary finding, we dichotomised observed CORE–OM scores during the examination period according to those above or below one total mean score point (appendix p 28), a threshold that discriminates between UK NHS clinical samples and general population samples.^28^ MSS participants were a third less likely than support as usual participants to be in this clinical range of distress (risk ratio 0·65, 95% CI 0·53–0·80; p<0·0001). On average, about six students (95% CI four to ten) needed to be offered the MSS course to prevent one from being distressed at a clinical level during the examination period.

The effect of the mindfulness intervention in participants who had examinations or assessments during the examination period (n=267) was on average 0·19 CORE–OM score points greater than the effect in those with no known assessments during this period (p=0·043; appendix p 8). The effect of the mindfulness intervention in men was on average 0·18 CORE–OM score points less than the effect in women, but did not differ significantly between sexes (p=0·061; appendix p 8).

**Figure 3:**
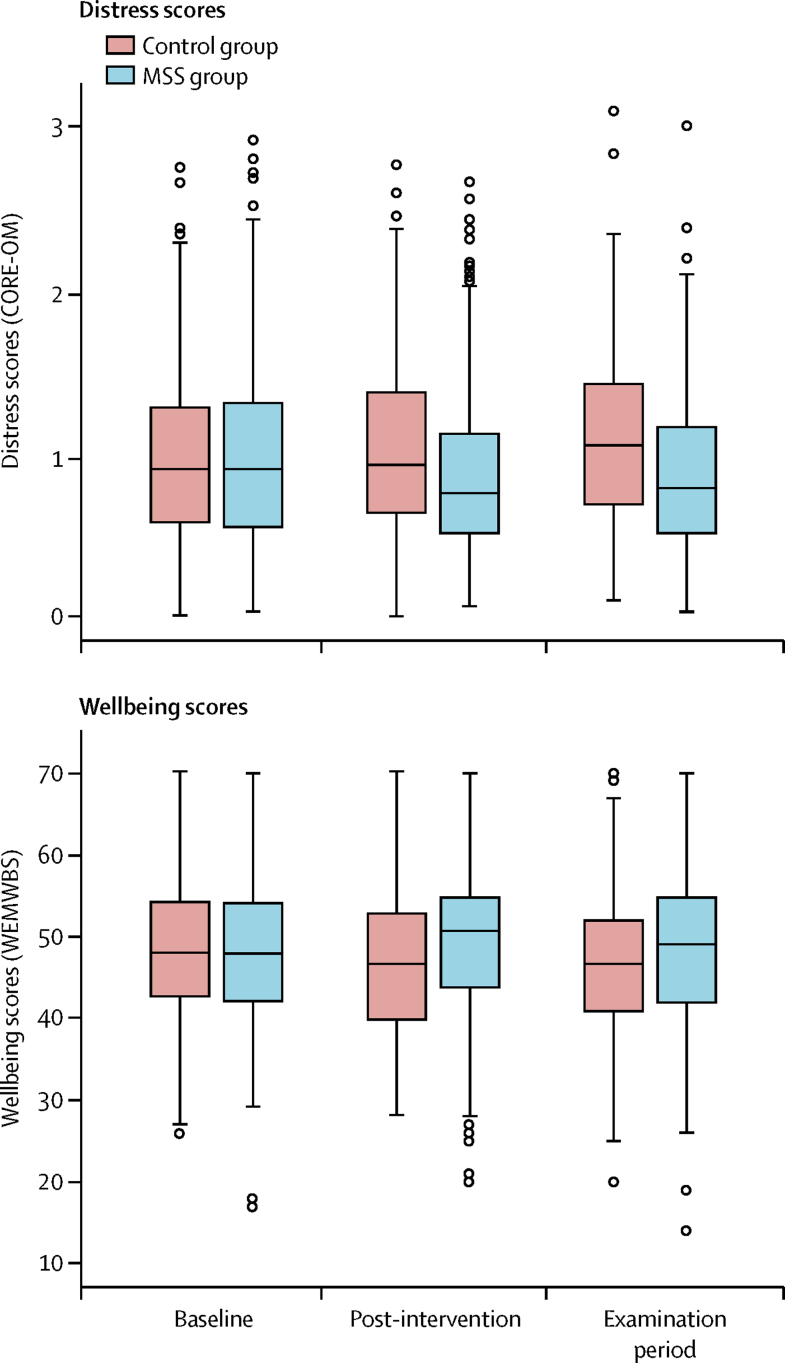
Distress and wellbeing scores Higher CORE–OM scores indicate greater distress and higher WEMWBS scores indicate greater wellbeing. Boxes show median values (middle lines) with 25th and 75th percentiles; whiskers denote values within 1·5 times of the IQR. Circles represent datapoints that fall outside this range. MSS=Mindfulness Skills for Students. CORE–OM=Clinical Outcomes in Routine Evaluation Outcome Measure. WEMWBS=Warwick–Edinburgh Mental Wellbeing Scale.

In analysis of secondary outcomes, mindfulness training reduced distress immediately after the course compared with support as usual (table 3). Distress among participants in the support as usual group increased over the academic year, whereas for MSS participants, a decrease in CORE–OM scores after the course was maintained during the examination period (figure 3, appendix p 27).

MSS improved wellbeing during the examination period and after the course compared with support as usual (table 3). Warwick–Edinburgh Mental Wellbeing Scale scores among support as usual participants decreased over the academic year, whereas for MSS participants, wellbeing increased after the course and was maintained during the examination period, although less so than CORE–OM scores (figure 3).

In the intention-to-treat population, students assigned to receive MSS were slightly more likely to get the highest (first-class) or lowest (third-class) grades than were those assigned to receive support as usual (no differences in the few fails; appendix p 15). Results for grade attainment were no longer statistically significant in a post-hoc sensitivity analysis in the per-protocol sample (ie, participants who completed at least four mindfulness course sessions), but between-group differences were numerically similar to those in the intention-to-treat analysis (appendix p 15). Participants with third-class grades (n=7, all in the MSS group) were no more distressed during the examination period than were higher-marked students (appendix p 30). The number of participants requesting special arrangements for their examinations did not differ between groups, nor did the number unable to sit their examinations (appendix pp 16, 17).

MSS participants reported fewer problems affecting either their study or university experience than did support as usual participants (appendix p 18), but there were no differences in how often they reported they would consider leaving their course, nor in their day-to-day coping with academic workload (appendix p 32). Post-intervention and during the examination period, more participants in the MSS group than the support as usual group donated their vouchers offered to recompense participation (appendix p 19).

Only 31 (5%) participants contributed to physical activity data due to technical limitations or their preference (appendix p 20). Activity in MSS participants (n=16) was reduced between 1400 and 1700 h (appendix p 31). This reduction cannot be attributed to afternoon meditation because none of these participants meditated for more than 1 h per week, and ten (63%) did less than 0·5 h per week.

For all outcome timepoints, 20 MSS participants and 25 support as usual participants triggered the adverse event reporting protocol by exceeding cutoff scores for CORE–OM risk subscales (appendix p 21). For adverse events triggered by other means, one participant left the mindfulness course because they felt it was unhelpful, bringing unwanted matters to the fore. This person was referred for counselling and subsequently reported to the trial that they remained positively disposed to mindfulness.

## Discussion

Our findings suggest that the MSS course maintains wellbeing and engenders resilience to accumulation of stress during the academic year, notably during the summer examination period—our primary outcome. Although the average baseline CORE–OM score of all participants was slightly higher than reported student norms (0·76 score points),^20^ scores for MSS participants at the time of maximum stress (examinations) had fallen towards those norms, whereas scores in the support as usual group indicated increasing distress. The primary outcome results were maintained across intention-to-treat and per-protocol analyses, implying effectiveness, even with only 59% of participants attending at least half the sessions. The moderate effect size translated into about five of 30 students in each group being prevented from indicating distress scores during the examination period at a clinically significant level (a number needed to treat of six). This finding suggests that mindfulness is more effective than other preventive interventions.^29^

Distress in the MSS group was lower beyond the period of the mindfulness courses and into the examination period than at baseline, which is consistent with mindfulness building psychological resilience to academic distress, as are results for perceived problems affecting study or experience. Wellbeing results, and interaction tests showing that the effect might be larger in people who had more stress, support this hypothesis.

Previous trials indicate that mindfulness courses shortly before university examinations might improve performance.^30^, ^31^ Our analysis of undergraduate examination results following MSS earlier in the year suggests a more nuanced effect: more students achieved either higher or lower grades after the MSS course. At a borderline level of statistical significance, we view these results of a planned secondary analysis with caution. However, the effect is consistent with the well known, inverted-U relationship between arousal and performance first described by Yerkes and Dodson.^32^ The value of such an effect is, inevitably, personal and beyond the resolution of the trial; for instance, an MSS participant achieving a low mark might, in fact, have intermitted their studies but for mindfulness training. Low statistical power for analysis of intermissions precluded further comment. Nevertheless, we believe that this finding warrants further study.

Results for comparisons of other secondary outcomes require caution. MSS participants more often donated their study payments to charity compared with participants in the support as usual group, in line with evidence that mindfulness promotes altruism.^33^ Results for daily coping strategies did not differ between groups, although small but relevant differences might have gone undetected in our design. The measurement of physical activity through mobile phone accelerometers was difficult to interpret because of the small, self-selected subsample; essentially, this element of the trial was unsuccessful. The finding that higher baseline wellbeing was related to non-response is puzzling, and might be a type 1 error.

This pragmatic trial has strengths and limitations. By contrast with most psychological intervention trials,^11^, ^23^ we actively monitored adverse reactions. The mindfulness course evaluated in this trial, in addition to its selection criteria and support structure, is unlikely to cause clinically relevant adverse reactions. Monitoring suggests that students learning mindfulness should be encouraged to discuss any concerns, unpleasant experiences, or adverse life events with their mindfulness teacher, and should be offered extra support.^34^

The trial participants were a subsample of roughly 20 000 total students self-selected on the basis of a personal interest in mindfulness and the setting of concerns over student mental health. However, mindfulness training appealed to a wide range of Cambridge University students in terms of socio-demographic characteristics. Participants were, on average, slightly more stressed at baseline than their peers who did not volunteer for the study. Our results replicate evidence from several smaller, mostly less rigorous, trials done across a range of higher education institutions, populations, and countries.^15^, ^35^, ^36^ Findings from all these trials indicate that 8 week mindfulness courses reduce distress among university students.^12^, ^13^, ^14^, ^16^ Therefore, we believe that our findings are generalisable to university students in the UK and other high-income countries. The slightly larger effect in women than men also replicates previous findings.^15^

To our knowledge, the Mindful Student Study is the largest randomised controlled trial to date assessing mindfulness training for students. The study robustly assessed the effectiveness of provision of a preventive mental health support service in real life, and was co-produced with stakeholders, making it immediately informative for policy making; however, it lacked an active control intervention beyond the wide range of standard support available to our students. Therefore, it was neither possible, nor was it our intention, to establish how participants' expectations influenced the results, including through a nocebo effect,^37^ whereby participants receiving support as usual expect symptoms to worsen; the relevance of peer and teacher support; and other non-specific factors associated with mindfulness training. Thus, our study is a confirmatory effectiveness trial based on evidence showing the efficacy of mindfulness interventions in different contexts; there are reasons to believe that our results are due, at least partly, to an effect specific to the mindfulness-based stress reduction techniques that were taught.^11^

We regard the 59% attendance at half or more of the mindfulness course sessions as a finding in and of itself in the context of a pragmatic effectiveness trial. The attendance rate could have represented a constraint on statistical power, but our study was designed to accommodate this. Loss to follow-up was moderate in both groups and, despite strenuous efforts to collect data, remains largely unexplained; these problems are common in mindfulness trials.^38^ However, we made best use of available data to impute missing values. A high proportion of participants are expected to be lost to follow-up in trials testing behavioural interventions that are neither prescribed nor essential for health, and reasons are expected to be difficult to collect.

The primary and most secondary outcomes were self-reported, and participants were not masked to group allocation, such that responses might have been influenced by their expectations. Only one mindfulness teacher delivered the intervention, which strengthens the internal validity of our results, but could restrict their external validity if the beneficial effects were partly attributable to the personal attributes of this particular individual.

Well conducted and adequately powered comparative effectiveness research is needed into preventive mental health interventions (eg, comparing mindfulness with a positive psychology preventive intervention). Early clinical evidence suggests that mindfulness effects might not differ from other interventions,^11^ but in preventive programmes, personal preferences, feasibility, and stigma might exert as much influence on real-life success as efficacy.

In conclusion, our study suggests that offering openly accessible mindfulness interventions aimed at the well student population, separate from specific mental health services, is a useful addition to robust clinical interventions delivered by university counselling services. The 8 week mindfulness course adapted for university students tested in this trial is an acceptable, feasible, and effective component of wider student mental health strategies. Public health increasingly favours interventions to promote mental wellbeing placed in settings such as educational institutions;^8^ therefore, whether our findings have a wider application merits further study.
