# Supplementary material for: A mindfulness-based intervention to increase resilience to stress in university students (the Mindful Student Study): a pragmatic randomised controlled trial
Source: Lancet Public Health. 2017 Dec 19;3(2):e72–81. doi: 10.1016/S2468-2667(17)30231-1 (PMC5813792; doi:10.1016/S2468-2667(17)30231-1)
Supplement: Supplementary appendix [file mmc1.pdf]

THE LANCET
Public Health

Supplementary appendix

This appendix formed part of the original submission and has been peer reviewed. We post it as supplied by the authors.

Supplement to: Galante J, Dufour G, Vainre M, et al. A mindfulness-based intervention to increase resilience to stress in university students (the Mindful Student Study): a pragmatic randomised controlled trial. *Lancet Public Health* 2017; published online Dec 18. http://dx.doi.org/10.1016/S2468-2667(17)30231-1.

Effectiveness of providing university students with a mindfulness-based intervention to increase resilience to stress: a pragmatic randomised controlled trial

Julieta Galante, Géraldine Dufour, Maris Vainre, Adam P Wagner, Jan Stochl, Alice Benton,

Neal Lathia, Emma Howarth, Peter B Jones

Supplemental Information

Contents

1. Methods: intervention details 2
2. Methods: multiple imputation procedure details 3
    2.1 Inclusion of variables in imputation model 3
    2.2 Specification of imputation algorithm 4
3. Methods: day-to-day coping data collection and analysis details 5
4. Methods: smartphone accelerometer (physical activity) data collection and analysis details 6
5. Results: intervention receipt details 7
6. Results: further analysis of the primary outcome 8
7. Discussion: demographic data comparisons with wider student populations 9
References 10
Supplemental Tables 11
Supplemental Figures 22

1. Methods: intervention details

The Mindfulness Skills for Students (MSS) intervention consists of a secular, face-to-face, group-based skills training programme based on the course book 'Mindfulness: A Practical Guide to Finding Peace in a Frantic World' (2), and adapted for university students to facilitate the application of mindful awareness to study, positive decision-making and relationships.

MSS summarises the different types of meditation taught in the course book (2), as three choices which can be made at any moment in a meditation (or beyond), depending on what we feel is needed at that moment for a kinder, wiser response to our experience (Supplemental Figure 1). Within the three choices, MSS places greater emphasis on certain areas of mindfulness practice and approach than the 'Frantic World' course:

1. Noticing moments of meta-awareness when they arise, pausing there, and exploring/appreciating that very moment (Supplemental Figure 2);

2. From the outset, encouraging students to choose their next point of attention 'in the flow', organically (whether the breath, body, sounds, thoughts, feelings, or a broader whole-body sense of being – as it feels natural or possible at the time), rather than necessarily coming back to the breath or body;

3. Encouraging students to choose freely whether to continue attending in the present moment at all, and consciously allowing into their meditation other states of mind and body (such as plans, day-dreams, sleepiness), to develop inner listening and presence (and avoid any implied value judgement of clear or focused states as preferable or 'better').

The primary component of MSS is thus compassionate self-knowledge and self-discovery, over time. It describes mindful attention as "being with" whatever enters our experience or awareness – with a growing ability to de-centre by acknowledging and "giving space" to whatever comes into the experience (Supplemental Figure 3). The emphasis on choice also fosters a growing confidence in making trustworthy choices both in meditation and beyond, alongside a more fluid and kindly understanding of what or who constitutes "I", the person making those choices. This mitigates students' perfectionist tendencies to judge experience negatively, or blame themselves for "getting it wrong" when less attentive or uncomfortable states enter into meditation, study or life, and increases their ability to tolerate all experience with an increasing comfort zone around difficult experience (so it becomes, "more and more acceptable not to be okay").

While the course book is rooted in Mindfulness-based Cognitive Therapy (2), MSS adopts the flexibility often associated with Mindfulness-based Stress Reduction (e.g. moving the raisin exercise to week two) (3). It also draws upon two other mindful modalities, Nonviolent Communication (Supplemental Figure 4) (4), and Focusing (5). These help students to explore feelings safely (e.g. in weeks four and five), to foster a kind, empathic inner relationship, to understand how this positively affects outer relationships (e.g. week 6), and to explore needs both met and not met by actions such as autopilot (week 1) and procrastination (week 7). Further details are available in the study protocol (1), and on request.

2. Methods: multiple imputation procedure details

Multiple imputation was used to address missing data taking account of [a] variables to be included in the protocol-based primary outcome analysis, [b] variables related or potentially related to non-response (in the primary outcome), and [c] variables that explained a significant amount of outcome variance (1, 6). The multiple imputation model was devised by a statistician (JS) who had no access to information on trial arm (i.e. no variable in the dataset enabled them to learn the outcome of randomisation).

2.1 Inclusion of variables in imputation model

The imputation model for the primary outcome took account of distress scores (CORE-OM) from other time points and routinely collected demographics (1).

Guidance on the inclusion of variables in the imputation model is available from existing literature (6). As a general rule, using every bit of available information yields multiple imputations that have minimal bias and maximal certainty (7, 8). This guidance implies that the number of predictors to be chosen should be as large as possible; indeed, including as many variables as predictors as possible (given collection and availability) makes missing-at-random more plausible. However, because data sets often contain several hundred variables it is clearly not feasible to include all these variables. A strategy for selecting subsets can be summarized as follows (6):

1. Include all variables that appear in the complete-data model, i.e., the model that will be applied to the data after imputation. Failure to do so may bias the complete data analysis, especially if the complete-data model contains strong predictive relations. This step is somewhat counter-intuitive, as it may seem that imputation would artificially strengthen the relations of the complete-data model, which is clearly undesirable. If done properly however, this is not the case. On the contrary, not including the complete-data model variables will tend to bias the results towards zero. Interactions of scientific interest should also be included into the imputation model.

   Accordingly, the following variables were included in the imputation model: Gender, Age, Trial Arm (outcome of randomisation), Cohort, and baseline CORE-OM scores.

2. Include variables that are related to the nonresponse. Factors that are known to have influenced the occurrence of missing data (stratification, reasons for nonresponse) are to be included on substantive grounds.

   Given this rule the following variables were identified to be related to non-response in the primary outcome based on expert opinion: Ethnicity, Disability, Financial help, Socio-economic status, Experience with meditation, College, School, Year of study programme, Type of consent, Degree, whether participant is in last year of study, Adverse event alarm at baseline and post intervention, Number of reminders sent at post intervention and examination period, Donation to charity at post intervention. Financial help and Socio-economic status variables were only used as auxiliary variables in this study (informed us about missing values in other variables). They were not used or reported elsewhere in the study because there was information for less than 50% of the sample due to the way the University of Cambridge registry routinely collects these.

3. In addition, include variables that explain a large amount of variance. Such predictors help to reduce the uncertainty of the imputations. They are crudely identified by a correlation above 0.5 with the target variable (CORE-OM at examination period) (9).

Wellbeing scale scores (WEMWBS) at all time points correlate strongly (though negatively) with CORE-OM at examination period. Therefore we have included WEMWBS scores in the imputation model to contribute to reducing uncertainty in imputations.

The final model for imputation therefore consisted of the variables named above.

2.2 Specification of imputation algorithm

Multiple imputation was performed using the R package *mice* (6) which adopts a chained equation algorithm. For numeric variables, predictive mean matching (pmm) was used (10). For categorical variables with two categories logistic regression (logreg) and for variables with more than two categories, a multinomial logit model (polyreg) was used. If categories were ordered, then an ordered logit model (polr) was used instead of multinomial logit.

Fifty imputations were performed using R 3.3.2 (11).

3. Methods: day-to-day coping data collection and analysis details

After completing the main examination period survey (which contained distress and wellbeing questionnaires), participants were invited to answer daily questions every morning for two consecutive weeks about how they had coped with academic matters the day before (the starting date would be depend on when the participant agreed to complete this part). They could choose to get a daily email with a link to an online questionnaire, or to use the Easy M Android-powered phone application (12) which would automatically trigger a phone notification and display the questions each day (http://nlathia.github.io/easym.html).

The problem-focused coping questions were ‘Did you study as much as you had planned yesterday?’ and ‘Did you take as many breaks from study as you had planned yesterday?’. The response item scorings (mutually exclusive options) for both questions were ‘Much less’=0, ‘Less’=1, ‘The same as planned’=2, ‘More’=1, ‘Much more’=0 (participants only saw the text, and not the scores). The rationale behind assigning the highest score to ‘the same as planned’ was that this is the best reflection of successfully coping by planning to solve a problem adequately, rather than making unfeasible/unbalanced plans (13).

The emotion-focused coping questions were ‘How satisfied with yourself are you about the amount you studied yesterday?’ and ‘How satisfied with yourself are you about the breaks from study you took yesterday?’ The response item scorings (mutually exclusive options) for both questions were ‘Very satisfied’=4, ‘Somewhat satisfied’=3, ‘Neither satisfied nor dissatisfied’=2, ‘Somewhat dissatisfied’=1, ‘Very dissatisfied’=0 (participants only saw the text, and not the scores).

The total coping score was calculated by adding the response values of these four questions. Nonparametric item-response theory analysis with a dataset devoid of arm data was used to check whether these scoring approaches were psychometrically valid (14).

Two further questions assessing motivational relevance (‘How motivated did you feel by academic matters yesterday?’ and ‘How stressed did you feel by academic matters yesterday?’; mutually exclusive response item scorings for both questions (‘Extremely’=4, ‘Very’=3, ‘Moderately’=2, ‘Slightly’=1, ‘Not at all’=0) were used to confirm that students cared about academic matters enough to be needing to elaborate coping strategies (13). This was done by adding up the scores of the first question with the inverse scores of the second question (participants only saw the text, and not the scores). It was confirmed that all students were on average more than moderately motivated and/or stressed (mean=4.3, standard deviation 0.94).

4. Methods: smartphone accelerometer (physical activity) data collection and analysis details

The Easy M Android-powered phone application (12) was configured to collect data for 15 days following explicit consent of study participants. In the background, this app passively collected smartphone built-in accelerometer data; the app was configured to sample this data every 15 minutes.

There are a number of factors that influence how much data we receive from each participant. These include: whether the participant left/logged out of or uninstalled the Easy M app, whether (and when) the participant has turned off his/her phone or it has turned off by running out of battery, and device-specific details that prevent data from being collected in the background. It is therefore common to have a variable amount of data per participant.

Each sample of raw data contained three time series: the acceleration that the device has sensed along the x, y, and z axes. To process this data into a score indicating how "active" the participant was, we first computed the magnitude vector. The magnitude of acceleration at a particular time t is defined as:

$m_t = \text{sqrt}(x_t^2 + y_t^2 + z_t^2)$

This vector indicates higher levels of activity if it has a high standard deviation (12). Therefore, we extracted an activity score by computing the standard deviation of the magnitude vector. Then, we examined the derived values at an aggregate level by arm of the study, by looking at the mean across all participants, binned by the time of day.

5. Results: intervention receipt details

Out of the 309 participants randomised to be offered the MSS course, 182 participants (59%) attended four or more sessions, and 65 participants (21%) attended all course sessions. Randomised participants attended a median of five sessions (inter-quartile range 2-7) including 44 who attended no sessions at all.

Being disabled reduced the odds of completing at least four sessions of the mindfulness course, set as minimum dose (see Supplemental Table 2). Numbers of sessions attended (Supplemental Table 3) suggest that participants dropped out from the courses, rather than just skipping sessions.

Thirty-nine MSS participants provided reasons for abandoning their mindfulness course: schedule conflicts (n=16) and being too busy (n=12) were the most frequent reasons. A few (n=15) cancelled without attending any sessions. Three people cancelled their place without attending any sessions due to misunderstanding eligibility or dates. Three participants who started the course found that it was not what they had expected (one did not like group sessions, one thought the groups were too big, one felt mindfulness' "roots in Eastern religion are not fully appreciated"). Two participants had emotional life events after starting the course and momentarily preferred to avoid developing their awareness of thoughts and feelings. Two participants cited personal reasons; they did not attend any course sessions. One participant found mindfulness momentarily unhelpful (see section 'Adverse events').

6. Results: further analysis of the primary outcome

As a sensitivity analysis, we calculated the primary outcome model without imputing missing values (i.e., using only observed data) and obtained very similar results (AMD -0.24, 95%CI -0.33 to -0.15, p<0.0001). A comparison of imputed main outcome values with observed main outcome scores shows that imputed distress scores are on average higher than observed scores, indicating that those who did not answer the questionnaire would have probably scored higher (see Supplemental Figure 8). As a further sensitivity analysis we calculated two-sample t-tests comparing trial arms using imputed and observed datasets and obtained similar results (p<0.0001 in all cases).

For our per protocol analysis, we also computed the primary outcome model with multiple imputation but only keeping MSS participants who completed at least half of the course (minimum dose) and both excluding individuals in the control group who engaged in meditation elsewhere during the follow-up period preceding outcome measurement (AMD -0.33, 95%CI -0.44 to -0.22, p<0.0001), and including them (AMD -0.31, 95%CI -0.41 to -0.21, p<0.0001), again obtaining very similar results.

As a way of benchmarking our results against scores more specific to university students, we further dichotomised observed CORE-OM scores using the average total mean score of UK students who attend student counselling services, which corresponds to 1.85 points (Supplemental Figure 7b) (15). MSS participants were almost two thirds less likely to be above the average distress of students needing counselling than SAU participants (RR 0.37, 95%CI 0.19 to 0.73). However, because few students score above the clinical average, 13 students needed to be offered the MSS course to prevent one student from being distressed above the clinical average during the examination period (NNT=13, 95%CI 8 to 38).

We conducted several sub-group analyses. All were pre-specified except for a post hoc analysis investigating the effect of cohort. None of them includes imputed data as we have not included some of the interaction variables in the multiple imputation for the primary outcome.

The effect of the mindfulness intervention on participants (N=267) who had examinations, PhD oral defences or thesis submissions during the examination period was on average 0.19 CORE-OM score points greater than the effect on those with no known assessments during this period (regression coefficient for interaction term (RCIT)=0.19, 95%CI 0.01 to 0.37, p=0.043). In determining if students had been assessed, we deviated slightly from the protocol: instead of using the recorded degree subject, we used assessment dates, because it was not possible to find out all participants' assessment dates based on their degrees. When it was not immediately clear that an assessment had taken place within the aforementioned period, we considered the case as unknown.

The effect of the mindfulness intervention on men was on average 0.18 CORE-OM score points smaller than the effect on women (RCIT=-0.18, 95%CI -0.37 to 0.01, p=0.061). There was no evidence of influence of baseline CORE-OM (RCIT<0.01, 95%CI -0.18 to 0.17, p=0.96), being a last-year student (RCIT=0.09, 95%CI -0.10 to 0.18, p=0.36), having prior meditation experience (RCIT=0.09, 95%CI -0.21 to 0.40, p=0.55), or being in a particular study cohort (RCIT=0.03, 95%CI -0.15 to 0.21, p=0.76) on the effectiveness of the intervention.

7. Discussion: demographic data comparisons with wider student populations

Demographic data shows that undergraduates and postgraduates volunteered for this study in proportions that reflect the Cambridge student population (16), as does the proportion of international students. Participants represented all University colleges and schools. Exam grades in the control group (Supplemental Table 5) are slightly better than those of all Cambridge students (27% first class, 57% upper-second class, 12% lower-second class, 2% third class, 1% other results) (17).

The proportion of women who volunteered (63%) reflects the percentage of gender breakdown for users of the University of Cambridge Counselling Service (UCS) and is higher than that in the student population (46%). The proportion of Asian students who volunteered (20%) is similar to the Cambridge student population and higher than that of UCS users (8.5%), which might reflect a lower stigma attached to mindfulness than to psychotherapy for this user group, and possibly a higher familiarity with meditation practices. The proportion of participants with disabilities (12%) is slightly higher than that of the Cambridge student population (8%), but lower than that of UCS users (14%).

At baseline participants reported more psychological distress than the general UK student population (total mean score=0.76, SD=0.59) (18), but lower levels than across those who attend university counselling services in the UK (total mean score =1.85, SD=0.51) (15). Our sample's mean score is just below the CORE-OM's recommended clinical cut-off score of 1 point, selected as a threshold to discriminate optimally between a clinical sample and a general population sample (19). Participants also reported lower wellbeing scores than the general student population (20).

These comparisons show that mindfulness training appealed to a wide range of University of Cambridge students who are slightly more stressed than their peers and perhaps at a higher risk of mental health problems, therefore more in need of help.

References

1. Galante J, Dufour G, Benton A, Howarth E, Vainre M, Croudace TJ, et al. Protocol for the Mindful Student Study: a randomised controlled trial of the provision of a mindfulness intervention to support university students' well-being and resilience to stress. BMJ Open [Internet]. 2016; 6. Available from: http://dx.doi.org/10.1136/bmjopen-2016-012300.
2. Williams M, Penman D. Mindfulness: a practical guide to finding peace in a frantic world: Hachette UK; 2011.
3. Kabat-Zinn J. Full Catastrophe Living, Revised Edition: How to cope with stress, pain and illness using mindfulness meditation. 2 ed. London: Piatkus; 2013.
4. Rosenberg MB. Nonviolent Communication: A Language of Life. 2nd ed. Encinitas CA: Puddledancer Press; 2003.
5. Gendlin ET. Focusing. New York: Bantam Books; 2007.
6. van Buuren S, Groothuis-Oudshoorn K. Mice: Multivariate imputation by chained equations in R. Journal of Statistical Software. 2011;45:1–67.
7. Meng X-L. Multiple-imputation inferences with uncongenial sources of input. Statistical Science. 1994:538-58.
8. Collins LM, Schafer JL, Kam C-M. A comparison of inclusive and restrictive strategies in modern missing data procedures. Psychological methods. 2001;6:330.
9. Cohen J. Statistical power analysis for the behavioral sciences. 2nd ed. Hillsdale, NJ: Lawrence Erlbaum Associates; 1988.
10. Little RJ. Missing-data adjustments in large surveys. Journal of Business & Economic Statistics. 1988;6:287-96.
11. R Core Team. R: A language and environment for statistical computing. Vienna, Austria: R Foundation for Statistical Computing; 2017; Available from: http://www.R-project.org/.
12. Lathia N, Sandstrom GM, Mascolo C, Rentfrow PJ. Happier People Live More Active Lives: Using Smartphones to Link Happiness and Physical Activity. PLoS One. 2017;12(1):e0160589. Epub 2017/01/05.
13. Smith CA, Kirby LD. Putting appraisal in context: Toward a relational model of appraisal and emotion. Cognition & Emotion. 2009;23(7):1352-72.
14. Stochl J, Jones PB, Croudace TJ. Mokken scale analysis of mental health and well-being questionnaire item responses: a non-parametric IRT method in empirical research for applied health researchers. BMC Med Res Methodol. 2012;12(1):1.
15. Stiles WB, Barkham M, Wheeler S. Duration of psychological therapy: relation to recovery and improvement rates in UK routine practice. [corrected]. Br J Psychiatry. 2015;207(2):115-22.
16. University of Cambridge. Student Statistics 2015-16. 2016 [10/07/2017]; Available from: http://www.prao.admin.cam.ac.uk/files/201516revise.pdf.
17. University of Cambridge. Examination Results and Degrees Statistics. 2015.
18. Core System Group. CORE system user manual. Leeds: Core System Group; Available from: http://www.coreims.co.uk/index.html.
19. Janice Connell MB, William B. Stiles, Elspeth Twigg, Nicola Singleton, Olga Evans, Jeremy N. V. Miles. Distribution of CORE–OM scores in a general population, clinical cut-off points and comparison with the CIS–R. British Journal of Psychiatry. 2006;190(1):69-74.
20. Stewart-Brown S, Janmohamed K. Warwick-Edinburgh mental well-being scale user guide. University of Warwick 2008 [10/07/2017]; Version 1 [Available from: http://www.mentalhealthpromotion.net/resources/user-guide.pdf].

Supplemental Tables

Supplemental Table 1. Regression model to assess baseline variables predicting missing primary outcome data (CORE-OM during the examination period).

	Odds Ratios	Standard Error	P value
Intervention arm	1.24	0.21	0.31
Cohort 1	0.98	0.21	0.91
Age	0.96	0.02	0.08
Gender – Men	0.71	0.21	0.11
Ethnicity – White	1.14	0.27	0.62
Nationality* – International	0.7	0.31	0.24
Disability – Yes	1.11	0.33	0.75
Degree – MPhil**	1.42	0.43	0.41
Degree – PhD**	1.25	0.41	0.59
Degree – Undergraduate**	0.67	0.4	0.31
Last Year	0.55	0.24	0.01
Previous meditation***	0.99	0.33	0.96
Baseline WEMWBS	0.95	0.02	0.01
Baseline CORE-OM	0.55	0.3	0.05

* Inferred from whether participants paid their study fees according to UK/European Union, or overseas rates
** Reference level: Degree – Masters
*** Spent in total more than 10 hours meditating in the past, or completed an eight-week mindfulness course.
Abbreviations: CORE-OM: Clinical Outcomes in Routine Evaluation Outcome Measure; WEMWBS: Warwick-Edinburgh Mental Wellbeing Scale.

Supplemental Table 2. Regression model to assess baseline variables predicting completing less than the minimum dose (4 sessions) of the mindfulness course in the intervention arm (N=309).

	**Odds Ratios**	**Standard Error**	**P value**
Cohort 1	1.08	0.27	0.77
Age	0.99	0.03	0.72
Gender – Men	0.94	0.28	0.82
Ethnicity – White	1.09	0.37	0.81
Nationality* – International	1.04	0.42	0.92
Disability – Yes	0.33	0.48	0.02
Degree – MPhil**	1.85	0.58	0.29
Degree – PhD**	1.56	0.57	0.44
Degree – Undergraduate**	1.01	0.52	0.97
Last Year	0.88	0.31	0.68
Previous meditation***	1.30	0.41	0.52
Baseline WEMWBS	1.00	0.02	0.94
Baseline CORE-OM	0.51	0.37	0.06

* Inferred from whether participants paid their study fees according to UK/European Union, or overseas rates
** Reference level: Degree – Masters
*** Spent in total more than 10 hours meditating in the past, or completed an eight-week mindfulness course.
Abbreviations: CORE-OM: Clinical Outcomes in Routine Evaluation Outcome Measure; WEMWBS: Warwick-Edinburgh Mental Wellbeing Scale.

Supplemental Table 3. Number of participants attending each session of the Cohort 1 (n=342) and Cohort 2 (n=274) mindfulness courses.

	**Cohort 1 courses**	**Cohort 2 courses**
Session 1	129	117
Session 2	125	95
Session 3	97	85
Session 4	91	82
Session 5	78	67
Session 6	80	67
Session 7	76	57
Session 8	66	57

Supplemental Table 4. Pooled regression estimates for distress during examination term with multiple imputation (primary outcome).

Adjusted R square = 0.20	**Regression coefficient (95%CI)**	**Standardised coefficient (β)* (95%CI)**	**P value**	**Lambda****
MSS arm	-0.25 (-0.34 to -0.16)	-0.44 (-0.60 to -0.29)	<0.0001	0.21
Baseline CORE-OM	0.41 (0.32 to 0.50)	0.39 (0.30 to 0.47)	<0.0001	0.30
Age	0.01 (0.00 to 0.02)	0.09 (0.00 to 0.18)	0.049	0.34
Gender - men	-0.03 (-0.12 to 0.01)	-0.05 (-0.22 to 0.12)	0.56	0.27
Cohort 1	0.10 (0.01 to 0.19)	0.18 (0.02 to 0.34)	0.029	0.20

*This is the regression coefficient expressed in standard deviations, rather than the variable's scale of units.
** Proportion of the total variance that is attributable to the missing data
Abbreviations: CI=confidence interval; CORE-OM=Clinical Outcomes in Routine Evaluation Outcome Measure; MSS: mindfulness skills for students.

Supplemental Table 5. Undergraduate participants attaining each examination grade.

Exam grades	Intention-to-treat analysis*		Per protocol analysis**	
	MSS<br>n (%)	SAU<br>n (%)	MSS<br>n (%)	SAU<br>n (%)
First-class	47 (32)	37 (26)	25 (31)	35 (26)
Upper second-class (2.1)	78 (53)	92 (64)	43 (53)	86 (63)
Lower second-class (2.2)	14 (9)	13 (9)	10 (12)	13 (10)
Third-class	7 (5)	0 (0)	2 (3)	0 (0)
Fail	2 (1)	2 (1)	1 (1)	2 (1)

* Fisher's Exact Test p=0.04
** Fisher's Exact Test p=0.27
Abbreviations: MSS=mindfulness skills for students; SAU=support as usual.

Supplemental Table 6. Special exam arrangements requests.

	**Due to any issue**		**Due to mental health issues**	
	MSS<br>n (%)	SAU<br>n (%)	MSS<br>n (%)	SAU<br>n (%)
No request	189 (89)	173 (85)	209 (99)	199 (98)
Request	23 (11)	30 (15)	3 (1)	4 (2)

Note: Includes all undergraduates and postgraduates where we have clear evidence of them being examined.
Abbreviations: MSS=mindfulness skills for students; SAU=support as usual.

Supplemental Table 7. Intermissions of study (i.e. students unable to sit exams).

	MSS n (%)	SAU n (%)
Not intermitted	304 (98)	304 (99)
Intermitted	5 (2)	3 (1)

Abbreviations: MSS=mindfulness skills for students; SAU=support as usual.

Supplemental Table 8. Perceived impact of problems on academic affairs.

Question	Response	MSS<br>n (%)	SAU<br>n (%)
Considering leaving course	Not at all	163 (69)	137 (65)
Test for differences between arms of the study:	Only occasionally	42 (18)	45 (21)
X^2=3.65, df=4, p=0.46	Sometimes	19 (8)	15 (7)
	Often	4 (2)	9 (4)
	Most of the time	7 (3)	6 (3)
Problems affecting study	Not at all	53 (22)	47 (22)
Test for differences between arms of the study:	Only occasionally	93 (39)	66 (31)
X2=10.26, df=4, p=0.04	Sometimes	42 (18)	53 (25)
	Often	38 (16)	27 (13)
	Most of the time	10 (4)	20 (9)
Problems affecting experience	Not at all	63 (27)	51 (24)
Test for differences between arms of the study:	Only occasionally	87 (37)	57 (27)
X^2=11.28, df=4, p=0.02	Sometimes	45 (19)	61 (29)
	Often	28 (12)	22 (10)
	Most of the time	13 (6)	21 (10)

Abbreviations: MSS=mindfulness skills for students; SAU=support as usual.

Supplemental Table 9. Participants donating their study payment to charity.

Time point:	Post-intervention*		Exam period**	
**Donated or retained (n (%)):**	**Donated**	**Retained**	**Donated**	**Retained**
SAU	100 (45)	124 (55)	94 (44)	120 (56)
MSS	156 (61)	99 (39)	138 (58)	98 (42)

*p=0.0004 for differences between trial arms.
** p=0.003 for differences between trial arms.
Abbreviations: MSS=mindfulness skills for students; SAU=support as usual.

Supplemental Table 10. Participants' agreement to smartphone data collection.

Response	n (%)
"No, I am not interested in answering these questions"	174 (42)
"Yes, I don't have a smartphone"	43 (10)
"Yes, I have a different smartphone"	117 (28)
"Yes, I have a smartphone with Android version 4.1 or newer"	62 (15)
"Yes, I have an Android smartphone but don't want movement sensor data to be collected"	17 (4)

Supplemental Table 11. Number of adverse events triggered by surpassing CORE-OM risk subscales cut-off scores.

Time point	MSS	SAU
Baseline	15	11
Post-intervention	13	13
Examination term	7	12

Abbreviations: CORE-OM=Clinical Outcomes in Routine Evaluation Outcome Measure; MSS=mindfulness skills for students; SAU=support as usual.

.

Supplemental Figures

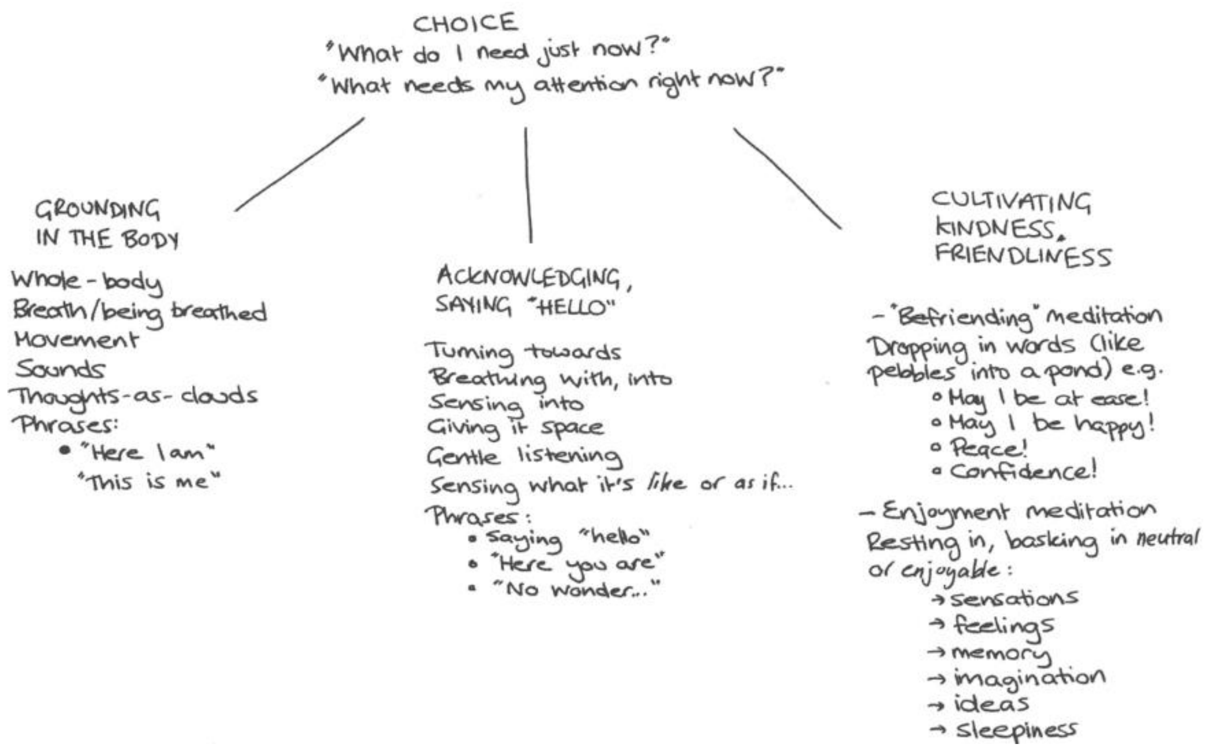

Supplemental Figure 1. Mindfulness Skills for Students course materials (see text for details).

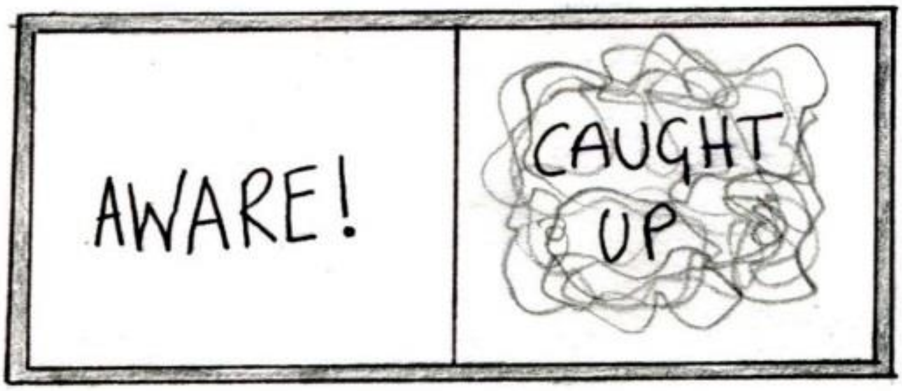

Supplemental Figure 2. Mindfulness Skills for Students course materials (see text for details).

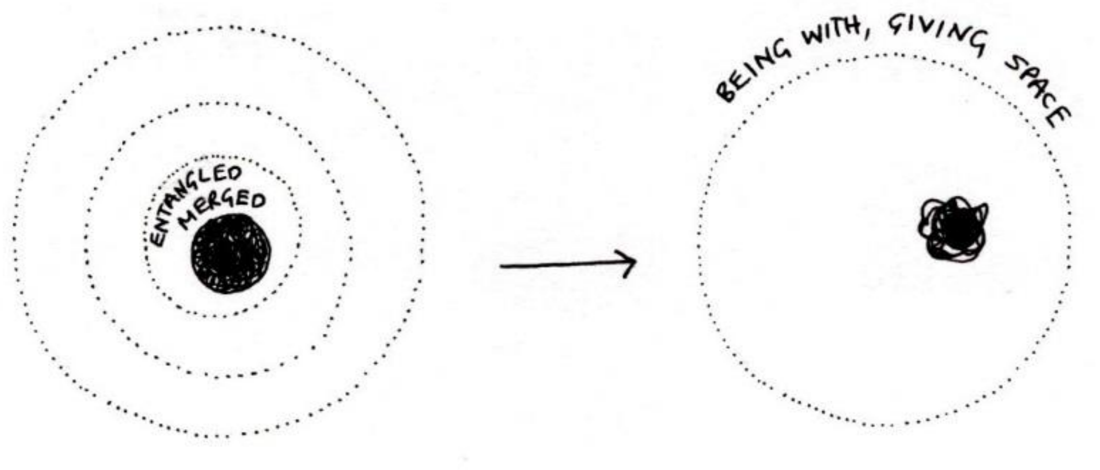

Supplemental Figure 3. Mindfulness Skills for Students course materials (see text for details).

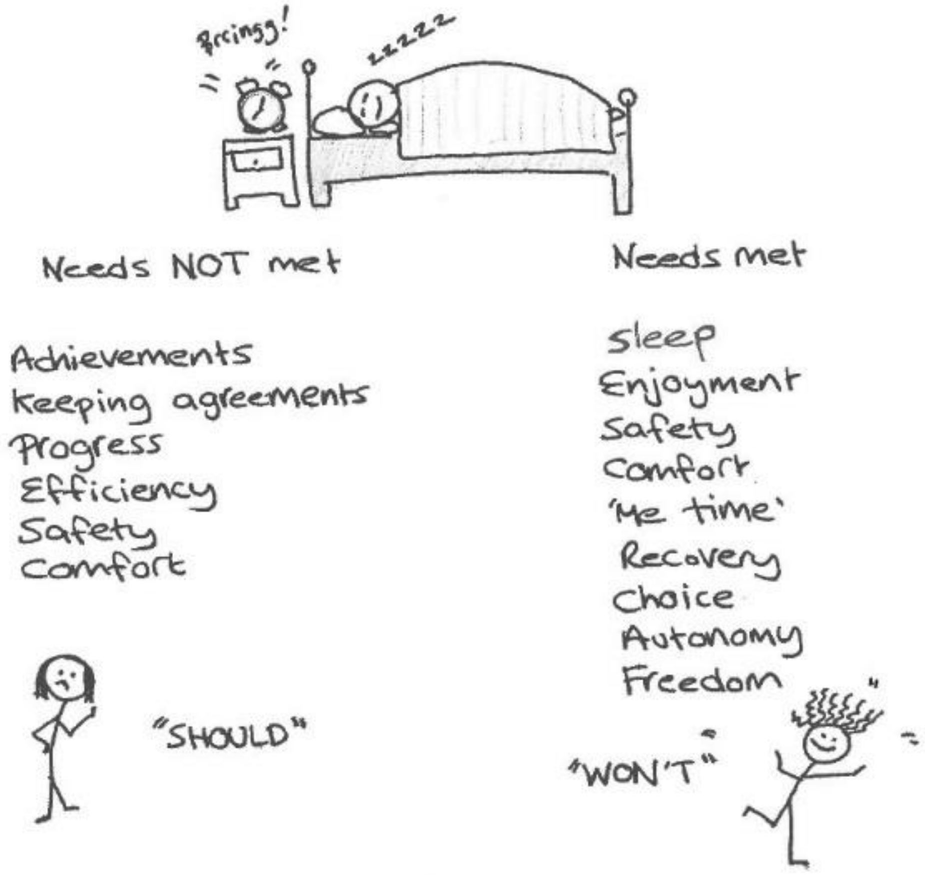

Supplemental Figure 4. Mindfulness Skills for Students course materials. MSS draws on Nonviolent Communication for a different way to approach balance and choices during the day: students suffer from guilt about what they have not done, and should do – so it is useful to look at their needs met and not met when they are procrastinating, removing the harsh judgement.

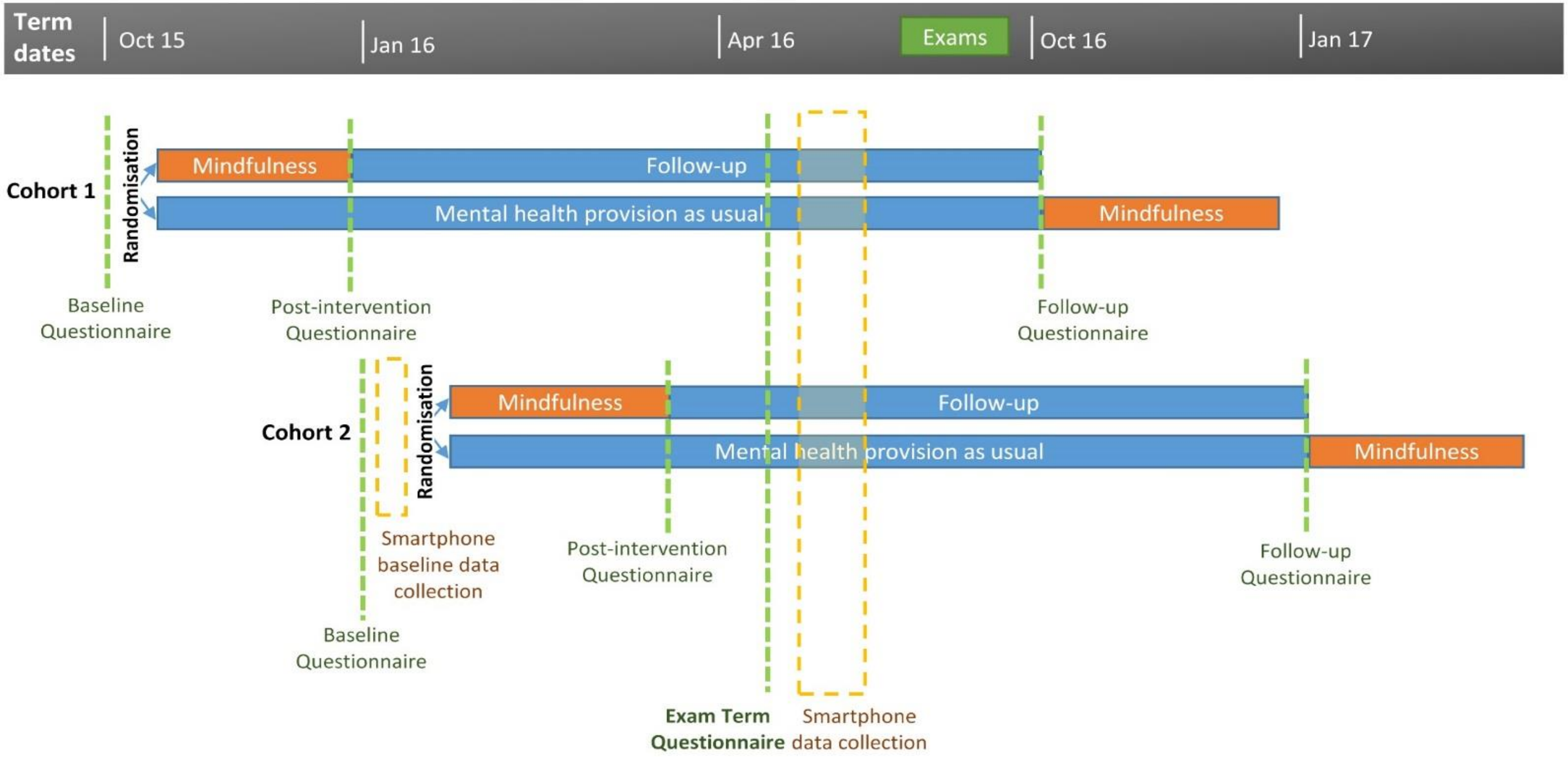

Supplemental Figure 5. Participant timeline (term dates bar not to scale). Source: Galante et al 2016 (1)

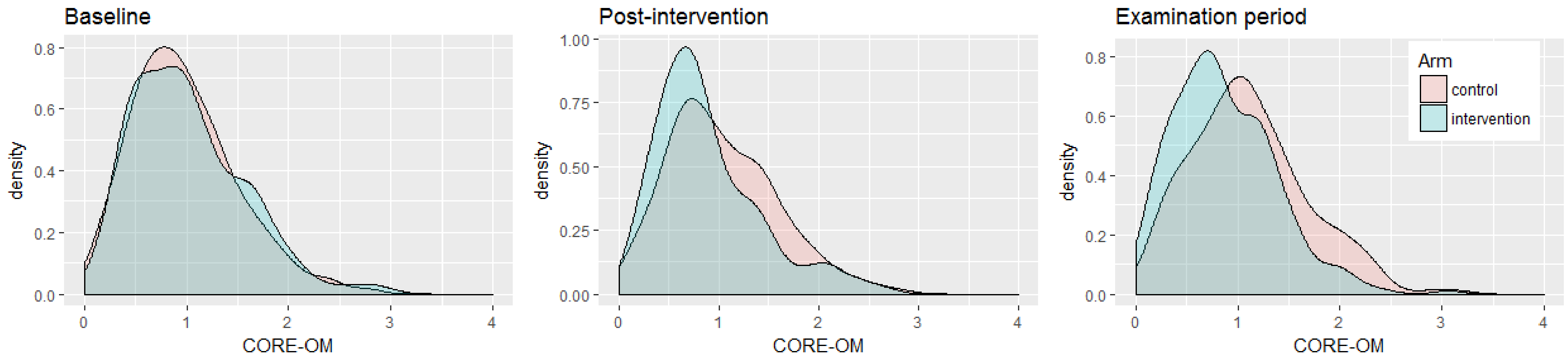

Supplemental Figure 6. Density plot illustrating the CORE-OM distress scores for each group (control in dark grey, mindfulness in light grey) as they progressed from the moment they signed up for the study (baseline), 2 months later (post-intervention) and during the examination period (1-2 terms later depending on whether students signed up in October 2015 or January 2016).

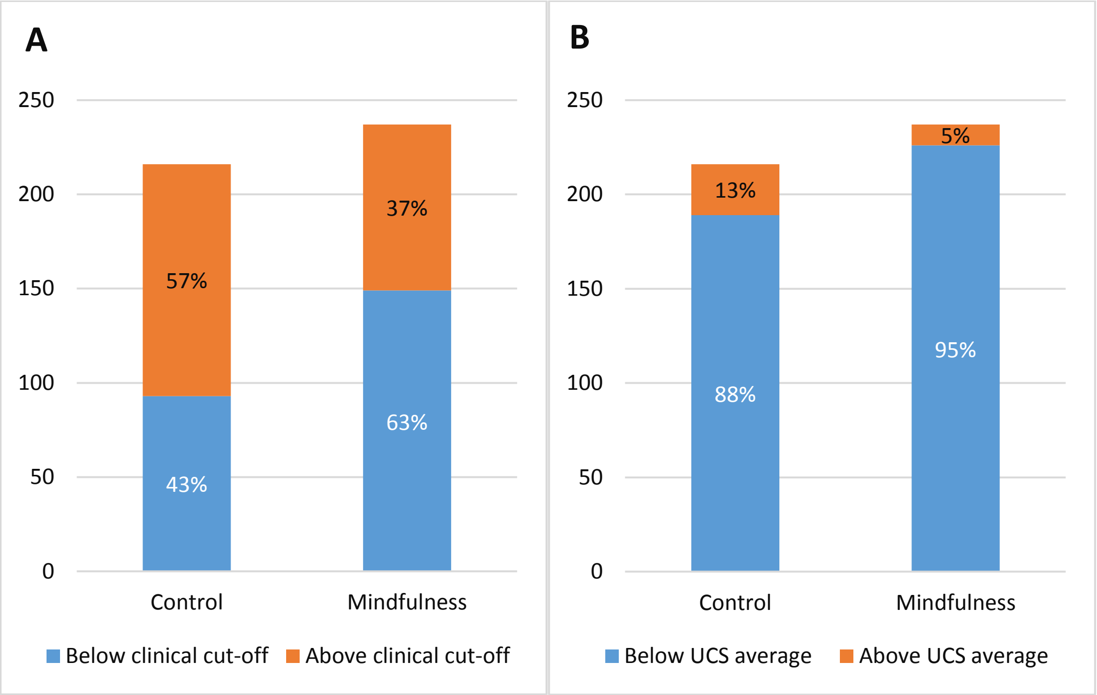

Supplemental Figure 7.Number of participants that during the examination period were above the CORE-OM validated clinical cut-off point for the UK general population (A), and above the CORE-OM average for UK students attending university counselling services (UCS) (B). Abbreviations: CORE-OM: Clinical Outcomes in Routine Evaluation Outcome Measure.

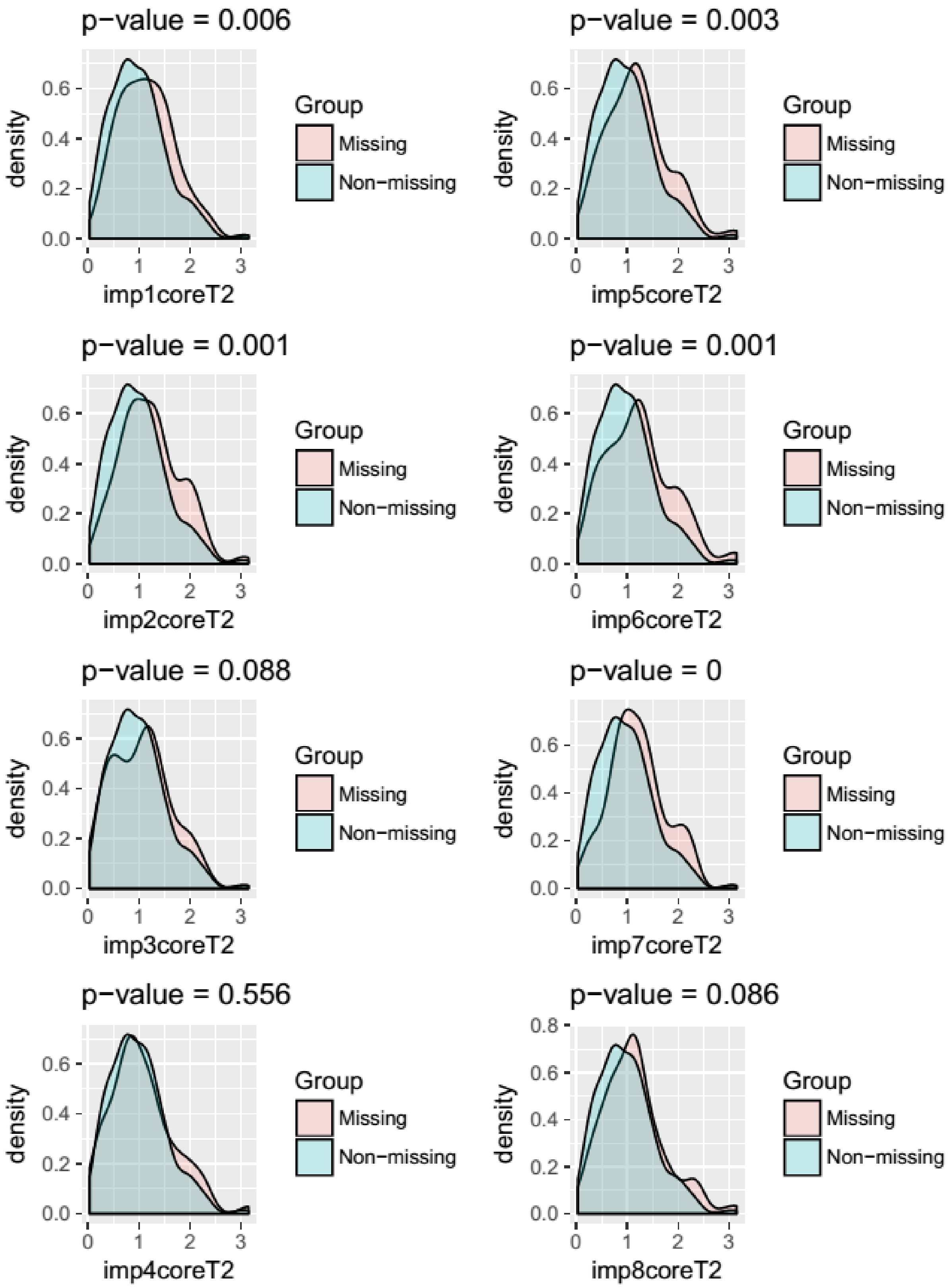

Supplemental Figure 8. Distribution of missing values that were imputed compared to non-missing values for the main outcome (exam period distress measured with CORE-OM), with p value for comparison (permutation test of equality of densities). Only the first eight imputed data sets are presented out of the 50 what were used for the multiple imputation of the main outcome.

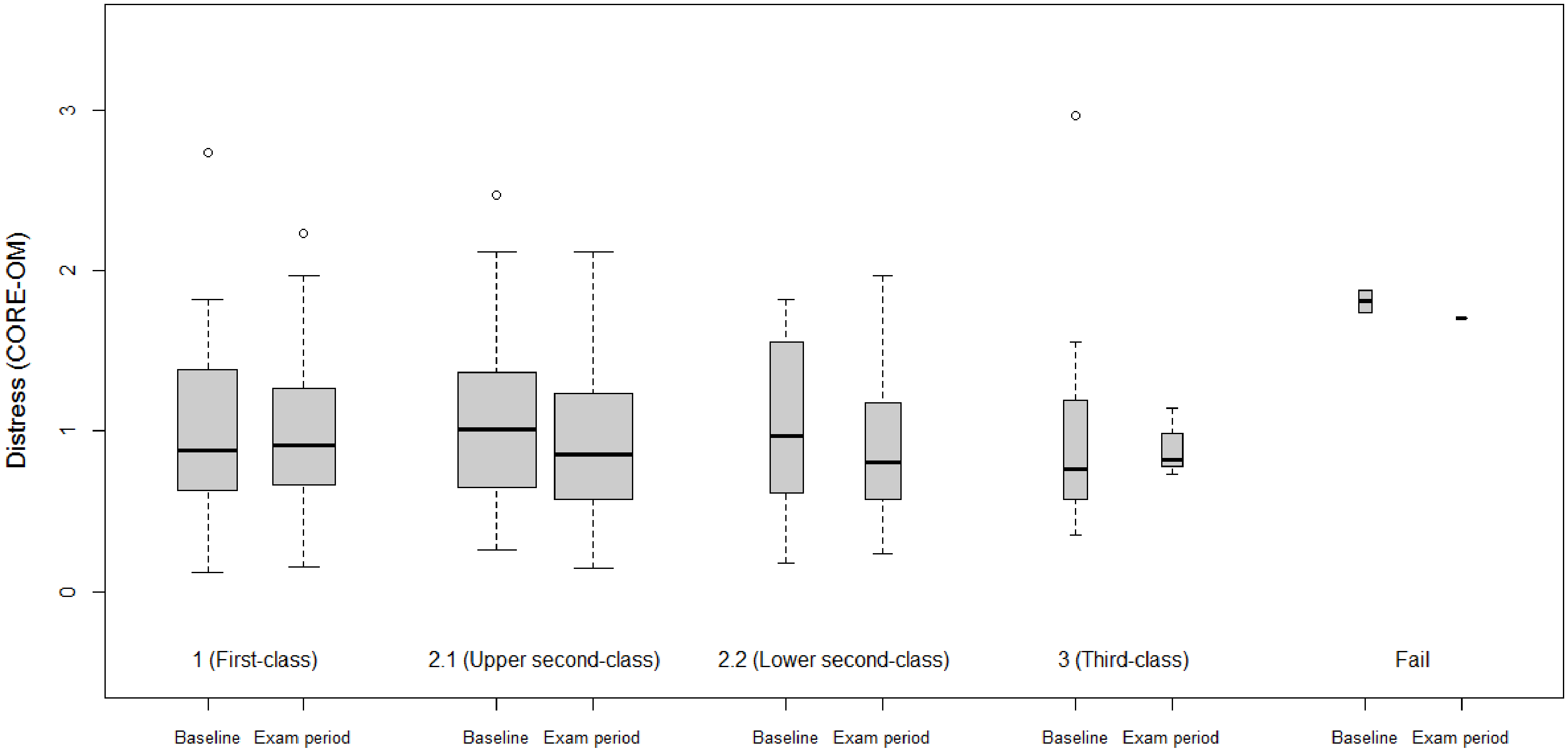

Supplemental Figure 9. Plot of psychological distress (CORE-OM) by exam grades. Box plots illustrating the distress scores for intervention participants by exam grades, as they progressed from the moment they signed up for the study (baseline), to the examination period (1-2 terms later depending on whether students signed up in October 2015 or January 2016); higher score indicates greater distress. The extent of the box is determined by the quartiles of the underlying data, with the middle line giving the median, and the whiskers include values within 1.5 times the interquartile range. Abbreviations: CORE-OM: Clinical Outcomes in Routine Evaluation Outcome Measure.

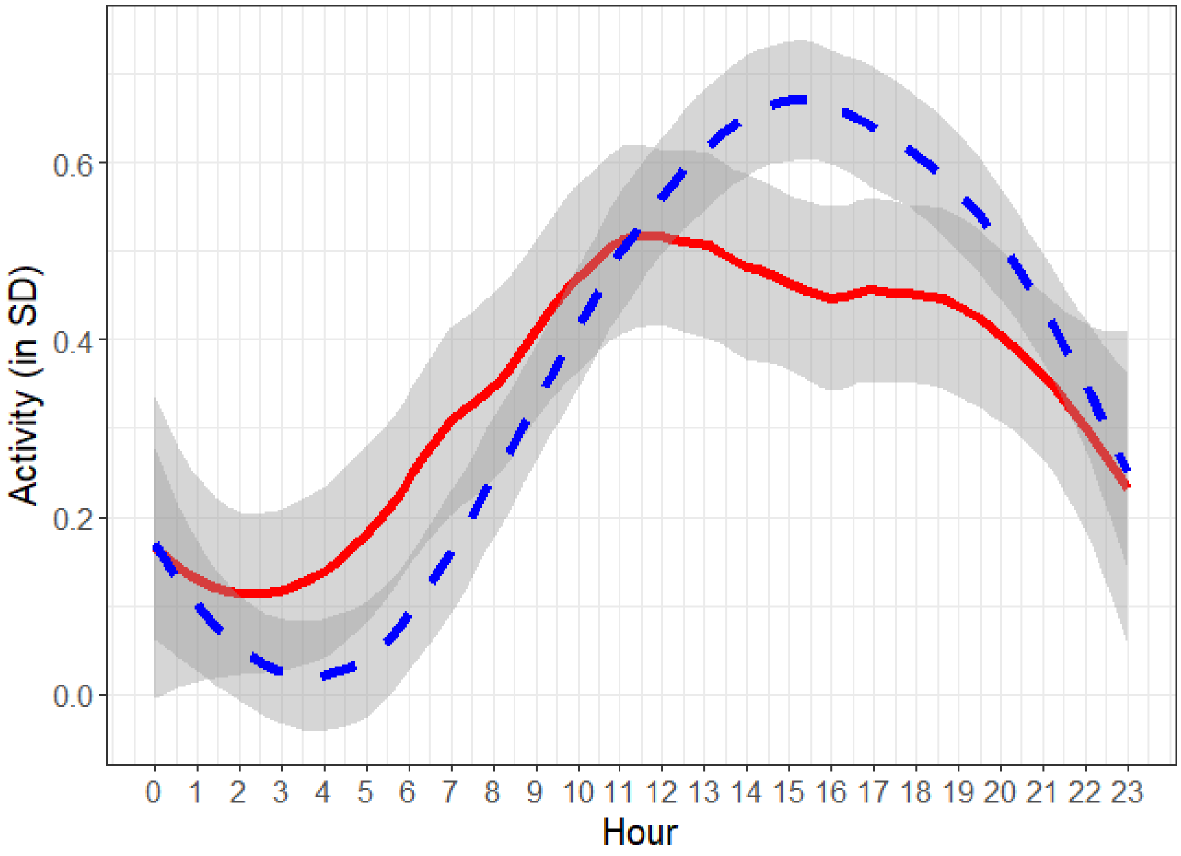

Supplemental Figure 10. Graph comparing the average activity per hour across all users in the control group (blue line), versus the average activity per hour across all users in the intervention group (red line). Shaded areas correspond to 95% confidence intervals around the respective average scores.

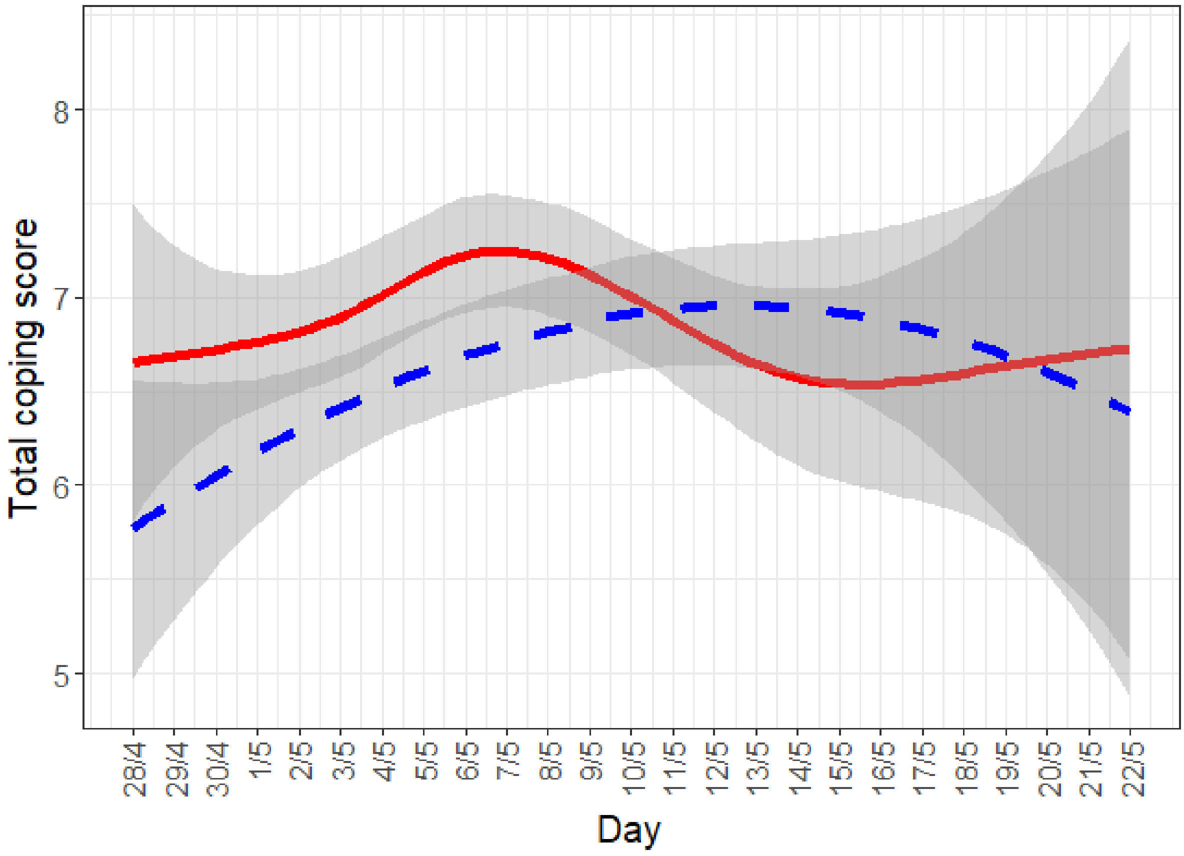

Supplemental Figure 11. Graph comparing the average coping score per day across all users in the control group (blue line), versus the average coping score per day across all users in the intervention group (red line). Shaded areas correspond to 95% confidence intervals around the respective average scores.
